# SBC Guidelines on Unstable Angina and Non-ST-Elevation Myocardial Infarction: Executive Summary

**DOI:** 10.5935/abc.20150118

**Published:** 2015-09

**Authors:** Gilson Soares Feitosa-Filho, Luciano Moreira Baracioli, Carlos José Dornas G. Barbosa, André Franci, Ari Timerman, Leopoldo Soares Piegas, José Antônio Marin-Neto, José Carlos Nicolau

**Affiliations:** 1 Hospital Santa Izabel da Santa Casa de Misericórdia da Bahia – Escola Bahiana de Medicina e Saúde Publica, Salvador, BA, Brazil; 2 Instituto do Coração (InCor/HCFMUSP), São Paulo, SP, Brazil; 3 Hospital do Coração, Brasília, DF, Brazil; 4 Instituto Dante Pazzanese de Cardiologia, São Paulo, SP, Brazil; 5 Faculdade de Medicina de Ribeirão Preto – USP, Ribeirão Preto, SP – Brazil

**Keywords:** Angina, Unstable / physiopathology, Myocardial Infarction / mortality, Troponin / therapeutic use

## Part I – Risk stratification and management within 12 hours of hospital arrival

### Introduction

Unstable angina (UA) is still one of the major cardiovascular causes of hospital admission. Some patients with UA develop elevations in biochemical markers of myocardial injury, characterizing myocardial infarction (MI) without ST-segment elevation (NSTEMI). Those two entities (UA and NSTEMI) make up the non-ST-elevation acute coronary syndromes (NSTE-ACS), the object of this guideline.

### Clinical history, physical examination and risk scores

**Figure 1:**
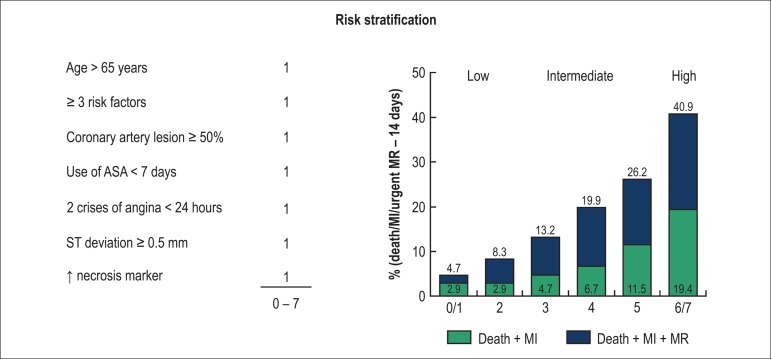
ASA: Acetylsalicylic acid; MI: Myocardial infarction; MR: Myocardial revascularization.

Clinical history and physical examination play fundamental roles in the risk stratification of patients with NSTE-ACS. The classification proposed by Braunwald, as well as its update, including troponin measurement, provide a rapid assessment of the patients' risk for major ischemic outcomes^1^. Mathematical tools, such as TIMI and GRACE scores, can provide prognostic information and guide risk stratification, as well as antithrombotic therapy^2,3^. (Figure 1 and Table 1)

**Table 1:** GRACE Score

**Risk stratification**				
Age (years)	- 0-100			
Heart rate	- 0-46			
Systolic blood pressure (mmHg)	- 58-0	**Risk**	**Score**	**% In-hospital death**
Creatinine (mg/dL)	- 1-28	Low	1-108	< 1
HF (Killip)	- 0-59	Intermediate	109-140	1-3
Cardiopulmonary arrest on admission	- 39	High	> 140	> 3
ST deviation	-28			
Elevation of necrosis markers	1 - 372			

HF: Heart failure.

The occurrence of major bleeding in patients with NSTE-ACS relates directly to adverse events (including mortality), and the use of bleeding scores (CRUSADE and ACUITY/HORIZONS), which estimate the risk of hemorrhagic complications, guide the therapy to minimize those outcomes^4,5^. (Tables 2 and 3)

**Table 2:** CRUSADE Score

**Prognostic factor**	**Scores**
**Baseline hematocrit (%)**	
< 31	9
31-33.9	7
34-36.9	3
37-39.9	2
> 40	0
**Creatinine clearance (mL/min)**	
< 15	39
16-30	35
31-60	28
61-90	17
91-120	7
> 120	0
**Heart rate (bpm)**	
< 70	0
71-80	1
81-90	3
91-100	6
101-110	8
111-120	10
> 120	11
**Sex**	
Male	0
Female	8
**HF signs on hospital arrival**	
No	0
Yes	7
**Previous vascular disease**	
No	0
Yes	6
**Diabetes mellitus**	
No	0
Yes	6
**Systolic blood pressure (mm Hg)**	
< 90	10
91-100	8
101-120	5
121-180	1
181-200	3
> 200	5

CRUSADE: Can Rapid risk stratification of Unstable angina patients Supress ADverse outcomes with Early implementation of the AmericanCollege of Cardiology/guidelines; HF: Heart failure. 1-20 very low risk (3.1%); 21-30 low risk (5.5%); 31-40 moderate risk (8.6%); 41-50 high risk (11.9%); 51-91 very high risk (19.5%).

**Table 3:** ACUITY/HORIZONS Score

													**Sum**
Sex						Men		Women					
						0		+8					
Age (years)	< 50			50-69			60-69			70-79		≥ 80	
	0			+3			+6			+9		+12	
Serum creatinine (mg/dL)	< 1		1-		1,2-		1,4-		1,6-		1,8-	≥ 2	
	0		+2		+3		+5		+6		+8	+10	
Total leukocyte count (giga/mL)	< 10		10-		12-		14-		1,6-		1,8-	≥ 20	
	0		+2		+3		+5		+6		+8	+8	
Anemia					No				Yes				
					0				+6				
ACS presentation		STEMI					NSTEMI				Unstable angina		
		+6					+2				0		
Antithrombotic agents					Heparin + GP IIb/IIIa inhibitors Bivalirudin								
						0		5				Total value	

ACS: Acute coronary syndrome; STEMI: ST-segment elevation myocardial infarction; NSTEMI: ST-segment elevation; GP: Glycoprotein. Algorithm used to determine the risk score for bleeding: < 10 low risk (1.9%); 10-14 moderate risk (3.6%); 15-19 high risk (6.0%); > 20 very high risk (13%).

#### Electrocardiogram

Despite its low sensitivity to discriminate a subendocardial MI from a transmural MI (by use of Q wave), the electrocardiogram (ECG) is fundamental to the management of patients with NSTE-ACS. Transient changes in the ST segment (depression or elevation), as well as T-wave inversions, are important prognostic markers of death or infarction. However, a normal ECG does not exclude the diagnosis of NSTE-ACS. It has prognostic importance, and the GUSTO II study has related initial ECG to early mortality as follows: left bundle branch block, left ventricular hypertrophy or pacemaker rhythm related to a mortality of 11.6%; ST-segment depression, mortality of 8%; ST-segment elevation, mortality of 7.4%; and T-wave inversion or normal ECG, mortality of 1.2%^6^.

#### Biochemical markers of myocardial necrosis

The modern biochemical markers (troponin and CKMB mass) are important tools for the diagnosis and prognosis of patients with NSTE-ACS. They should be interpreted in association with clinical and ECG findings, considering that several non-coronary conditions can determine their elevation^7^.

After percutaneous coronary intervention (PCI) or coronary artery bypass grafting (CABG), elevations in the levels of necrosis markers 5 and 10 times their reference values (post-PCI and post-CABG, respectively) indicate MI when interpreted in association with symptoms, ECG changes and/or imaging tests^8^. Myoglobin and high-sensitivity troponins, due to their high negative predictive value 6 hours after symptom onset, can be considered in protocols of early discharge from the emergency unit^9^.

#### Exercise testing

Patients with NSTE-ACS should undergo exercise testing (ET) with the following purposes: to identify occasional myocardial ischemia, to estimate prognosis and to guide proper clinical decisions, such as treatment strategies. It is recommended to patients at low risk as a first choice test, because it is a low-cost, low-risk, widely available procedure. A negative ET correctly indicated to a patient with good functional capacity allows immediate hospital discharge, because the test has a high negative predictive value^10^.

#### Echocardiography

Echocardiography is extremely useful in patients with NSTE-ACS^11^. The detection of changes in segmentary contraction strongly indicates coronary artery disease (CAD), because it can represent infarction, ischemia or both. In addition, it plays an important role in the differential diagnosis of chest pain (aortic dissection, aortic stenosis, pulmonary embolism, hypertrophic cardiomyopathy and pericardial disease) and in prognostic assessment via left ventricular ejection fraction (LVEF).

#### Nuclear cardiology

For patients with NSTE-ACS, the prognostic role of the information provided by nuclear imaging (myocardial perfusion and ventricular function) has been well established. Physical or pharmacological stress myocardial perfusion imaging is performed in low- to intermediate-risk patients with NSTE-ACS after stabilization of acute findings (48/72 hours). In the emergency unit setting or in the presence of acute pain, the radiotracer should be injected only at rest, while the patient is still symptomatic (exceptionally after the end of the symptoms), and the images should be obtained in up to 6 hours. If considered to be of low risk, it indicates a very low likelihood of subsequent cardiac events^12,13^.

#### Coronary computed tomography angiography

Coronary computed tomography angiography is an important tool to assess patients with acute chest pain, especially those at low and intermediate risk. It is safe for the diagnosis of NSTE-ACS and can reduce the length of hospital stay, and, thus, total cost.

#### Criteria for hospital discharge of low-risk patients in the first 12 hours of stratification

The criteria are lack of pain, clinical stability, normal ECG or ECG with no acute change, normal levels of myocardial necrosis biochemical markers, and negative provocative test of coronary spasm, when performed.

## Part II – Management of intermediate- and high-risk patients

### Admission to and discharge from the coronary care unit

All intermediate- and high-risk patients with NSTE-ACS should be admitted to the coronary care unit (CCU) whenever possible. Those undergoing PCI should return to the CCU after the procedure; in the absence of complication, they should be discharged from the CCU on the following day. When CABG is the treatment option, the patient should remain at the CCU up to surgery time. Those undergoing exclusive clinical pharmacological treatment should be discharged from the CCU on the day following that decision, provided they are stable and require no intravenous drug.

### Oxygen therapy

Limited and old evidence suggests that oxygen administration can limit the extension of acute ischemic injury^14^. Usually oxygen supplementation is maintained for up to 4 hours after pain subsides. If hypoxia persists, oxygen supplementation will be kept according to clinical need. Unnecessary oxygen administration for prolonged time can cause systemic vasoconstriction and even be harmful.

### Analgesia and sedation

The chest pain and anxiety of NSTE-ACS usually lead to hyperactivity of the sympathetic nervous system. In addition to increasing myocardial oxygen consumption, that hyperadrenergic state predisposes to atrial and ventricular tachyarrhythmias. Thus, strong analgesic drugs are recommended to patients with severe ischemic pain, who are refractory to antianginal therapy^15^. When pain is not relieved with sublingual nitrate, or when pain recurs despite adequate anti-ischemic therapy, morphine sulfate is the analgesic of choice, and should be intravenously administered, diluted at the dose of 2 to 4 mg every 5 minutes up to 25 mg, with blood pressure monitoring.

### Nitrates

The use of nitrates is based on their mechanism of action and clinical experience over several years of use. No controlled clinical trial has tested the effects of nitrates on clinical outcomes and mortality in UA, although their use is universally accepted^16,17^. The treatment is initiated at the emergency room, with sublingual administration of nitrate. If no rapid relieve of the pain occurs, those patients can benefit from the intravenous administration of nitroglycerin. Nitrates are contraindicated in the presence of important arterial hypotension (systolic blood pressure < 100 mmHg) or previous use of sildenafil in the past 24 hours. The intravenous treatment should be maintained for 24-48 hours after the last episode of anginal pain, and suspended gradually.

### Beta-adrenergic blockers

By decreasing heart rate, blood pressure and myocardial contractility, betablockers reduce myocardial oxygen uptake. Despite the lack of large-scale randomized studies assessing their action on major clinical outcomes, such as mortality, these drugs, along with nitrates, are considered first-choice agents in the treatment of NSTE-ACS^18^.

They should be initiated orally for stable patients with no contraindication, at low doses, which should be gradually increased to maintain heart rate around 60 bpm. If ischemic pain and/or tachycardia (not compensatory for heart failure) persists, the intravenous formulation can be used.

### Calcium channel blockers

The beneficial effects of calcium channel blockers on NSTE-ACS derive from the combination of their actions, reducing myocardial oxygen uptake, afterload, contractility and heart rate, in addition to increasing myocardial oxygen supply via coronary dilatation. Calcium channel blockers can be used to control refractory ischemic symptoms in patients already on adequate doses of nitrates and betablockers, or in those who do not tolerate those drugs (mainly those with contraindication), or those with variant angina (Prinzmetal syndrome). In patients with impaired left ventricular function and/or atrioventricular conduction changes, calcium channel blockers should be avoided^19^.

#### Inhibitors of the renin-angiotensin-aldosterone system

There is no conclusive evidence about the benefits of the early use of renin-angiotensin-aldosterone inhibitors to patients with NSTE-ACS, although some studies have suggested they can be useful in the chronic phase^20^.

### Antiplatelet agents

#### Acetylsalicylic acid

Coronary thrombosis plays an important role in NSTE-ACS triggering and progression, antithrombotic therapy being thus essential for patients with those syndromes. Acetylsalicylic acid (ASA) is the best antiplatelet agent, and should always be prescribed, except for the rare cases of previously known severe allergic reaction and the existence of active digestive bleeding^21^.

#### Thienopyridine derivatives

Thienopyridine derivatives are antagonists of the platelet activation mediated by the platelet adenosine diphosphate (ADP) receptor (P2Y_12_).

Clopidogrel in addition to AAS to treat intermediate-to high-risk NSTE-ACS was related to a 20% reduction in the risk of cardiovascular death, MI or stroke^22^. When the dose was doubled (600 mg loading dose, followed by 150 mg daily for 6 days), a 14% reduction in the risk of cardiovascular death, MI or stroke was observed in patients undergoing PCI (NNT, 167). However, a 41% increase in the occurrence of severe bleeding was observed (NNH, 200)^23^. Because the NNT to avoid an ischemic event is similar to the number of patients treated who will have a severe hemorrhagic event, careful and individualized assessment is recommended for each case.

The platelet aggregation inhibition of patients on clopidogrel has shown a wide intra- and interindividual variation. "Poor response" and "resistance" to clopidogrel are terms used to characterize patients who do not reach the expected platelet inhibition level. Consistent data have associated poor response to clopidogrel to the greater incidence of thrombotic events, mainly in patients undergoing PCI with stent implantation. Several strategies have been tested to decrease resistance to clopidogrel, but no significant positive impact has been observed on the reduction of clinical events^24^.

The association of clopidogrel with proton pump inhibitors (PPI), mainly omeprazole, has been related to a higher incidence of resistance to clopidogrel. Despite conflicting data, the routine use of PPI should be avoided and histamine H_2_ receptor antagonists should be preferred^25^.

Prasugrel is a third-generation thienopyridine derivative, which provides greater and more consistent platelet inhibition levels. Compared to clopidogrel when used for patients with high-risk acute coronary syndrome (ACS) undergoing PCI, prasugrel was associated with a 19% reduction in the occurrence of cardiovascular death, MI or stroke. However, the use of prasugrel was associated with a 32% increase in the occurrence of severe bleeding. Prasugrel should not be prescribed to patients with previous transient ischemic attack (TIA) or stroke. Its use in patients >75 years or <60 kg should be individualized^26^.

For patients undergoing CABG, clopidogrel should be suspended at least 5 days before the procedure, while prasugrel should be suspended 7 days before.

#### Cyclopentyl-triazolo-pyrimidine

Ticagrelor is a cyclopentyl-triazolo-pyrimidine (CPTP) with a 12-hour half-life, which, unlike thienopyridine derivatives, exerts a reversible block of P2Y_12_ receptors and whose action does not depend on liver metabolism. With such characteristics, ticagrelor has a more intense, rapid and consistent antiplatelet effect as compared to clopidogrel. In patients with intermediate- and high-risk ACS, as compared to clopidogrel, ticagrelor was associated with a significant 16% reduction in the occurrence of combined outcome of vascular death, MI or stroke. In addition, a 21% reduction in vascular deaths and a 22% reduction in all-cause mortality occurred. No significant increase in major hemorrhagic events, fatal bleeding or need for transfusion has been reported with ticagrelor; however, an increase in major bleeding not related to CABG has been reported. In addition, the use of ticagrelor has been associated with a higher incidence of dyspnea and transient ventricular pauses, as well as with an increase in creatinine and uric acid levels. In patients undergoing CABG, ticagrelor should be suspended 5 days before the procedure^27^.

#### Glycoprotein IIb/IIIa receptor inhibitors

That class of drugs blocks the common final pathway of platelet aggregation, regardless of the initial stimulus. By inhibiting glycoprotein (GP) IIb/IIIa receptors on platelet surface, those inhibitors prevent fibrinogen from binding to activated receptors, blocking platelet aggregation and platelet thrombus formation.

In the context of patients with NSTE-ACS undergoing an essentially "conservative" strategy, GP IIb/IIIa inhibitors have their use supported by studies on heparinization plus ASA^28,29^. Despite their extremely heterogeneous results, usually suggesting benefits deriving from the use of small-molecule GP IIb/IIIa inhibitors, but not from abxicimab, a meta-analysis^30^ has shown an only 9% reduction in the relative risk of death or infarction at 30 days of follow-up (p = 0.015), the benefit being restricted to higher-risk patients (high troponin and/or ST-segment depression and/or undergoing PCI).

Patients on oral dual antiplatelet therapy conducted via an early invasive strategy, GP IIb/IIIa inhibitors can be initiated in the catheterization laboratory, in the presence of high complexity PCI, high thrombotic load, no-reflow phenomenon or multiple instability sites of atherosclerotic plaques. GP IIb/IIIa inhibitors should always be used in an individualized and non-routine way.

### Antithrombin agents

Antithrombin therapy should be administered to all moderate and high-risk patients with NSTE-ACS, except when contraindicated. Low-molecular-weight heparins (LMWH) are usually as effective as unfractionated heparin (UNH); however, enoxaparin appears to be superior to UNH^31,32^. Patients receiving enoxaparin to treat NSTE-ACS and referred for PCI within 8 hours from the last subcutaneous dose require no additional anticoagulation. Those undergoing PCI between 8 and 12 hours should receive an additional intravenous dose of 0.3 mg/kg right before the procedure. The initially used heparin should be maintained during the entire heparinization period, avoiding the concomitant or alternate use of LMWH and UFH. Fondaparinux has demonstrated equivalence with enoxaparin to reduce ischemic events, being associated, however, with a significant reduction in severe bleeding^33^.

### Diagnosis and risk stratification with complementary tests

Risk stratification should be a continuous process, from initial clinical assessment, passing by subsidiary tests already discussed in this guideline, and culminating in the complementary tests described below.

Currently there is consistent evidence on the benefit of early "interventional" or "invasive" strategy for NSTE-ACS, aimed at performing coronary angiography usually within 24 hours from admission^34^. It is worth noting that the benefit observed with the "interventional" strategy tends to be greater in the long run than in the initial phase, in which, paradoxically, the risk of using that strategy can be higher^35^. In addition, the higher the risk for ischemic events, the more benefit there is. Furthermore, the appropriate antithrombotic regimen with antiplatelet and antithrombin agents is fundamental for the success of that approach.

#### Hemodynamic and cineangiocardiographic assessment

It essentially provides direct visualization of the coronary lumen, with assessment of the severity of obstructions, and analysis of the systolic and diastolic ventricular, global and regional functions. In addition, it can assess the functional meaning of anatomically detected lesions, by direct measurement of the coronary fractional flow reserve (FFR). However, it is worth noting that, in the NSTE-ACS context, that complementary test has limited applicability, because of the intrinsic mutability of the obstructions (often ulcerated, complex atherosclerotic plaques with high thrombotic load), and has not been duly validated in proper studies.

#### Exercise testing

Exercise testing can be the initial risk stratification approach for patients with NSTE-ACS when other non-invasive methods are unavailable and there is no indication for invasive strategy. In addition to diagnostic support, it has a well-known prognostic value. Positive tests are associated with a higher incidence of coronary events within 1 year as compared to negative tests. It is an inexpensive, safe procedure of easy application. Its negative predictive value is very high, 98% to 100%, although its positive predictive value is modest, around 50%. The ET, aimed at estimating prognosis and supporting clinical decision, is mainly indicated for intermediate-risk patients who can perform it 24 to 48 hours after complete clinical stabilization (hemodynamic stability, absence of active clinical or electrocardiographic ischemia, of new Q waves, of clinical signs of heart failure and normal markers of myocardial necrosis), as long as there is physical capacity. The ET should be carried out on a treadmill or cycle ergometer at a hospital, and always be symptom-limited^36^.

#### Echocardiographic assessments

##### Stress echocardiography

Stress echocardiography allows the assessment of transient regional abnormalities of contractility, indicative of induced ischemia. Pharmacological stress with dobutamine is safe and effective in that context, and provides prognostic information. However, the same restrictions and contraindications reported for ET apply for stress echocardiography. The following responses indicate higher risk: incapacity to increase EF or an EF reduction > 5% on exertion and regional contractility abnormality during stress. A segmental contractility improvement in dyssynergic areas with initial dobutamine doses (5 to 10 mg/kg/min) identify myocardial viability in those regions "stunned" by previous ischemia.

##### Studies with myocardial perfusion assessment

The development of contrast media containing smaller microbubbles of higher stability, in association with technological advances, such as intermittent harmonic imaging and low-mechanical index imaging, has allowed the echocardiographic study of myocardial perfusion. The use of contrast media during dobutamine stress echocardiography with real-time imaging analysis provides a simultaneous assessment of myocardial perfusion and of segmental motility changes.

#### Nuclear medicine methods

##### Myocardial perfusion imaging

Myocardial perfusion imaging (MPI) and radionuclide ventriculography play a significant role in the diagnosis and prognosis of ACS. Myocardial perfusion imaging is mainly indicated for patients who cannot undergo ET and those whose adequate interpretation of exercise ECG is difficult. Patients diagnosed with NSTE-ACS and having a normal MPI during stress belong to a subgroup with reduced risk of severe events, around 1% in one year. On the other hand, the detection of reversible defects expresses an unfavorable prognosis, with an event rate of 20% in the same follow-up period.

##### Radionuclide angiocardiography

Radionuclide angiocardiography is obtained by synchronizing the computed tomography scan with ECG (gated SPECT). It allows assessing regional systolic function and estimating ventricular EF, adding diagnostic and prognostic information.

##### Cardiovascular magnetic resonance

Cardiovascular magnetic resonance (CMR) can provide accurate information on heart morphology, volume quantification, global and regional ventricular mass and function. It allows assessing myocardial ischemia, by analyzing segmental contractility under dobutamine stress and without contrast or via myocardial perfusion under stress with vasodilators, such as dipyridamole or adenosine, and using gadolinium. In addition, it allows the assessment of myocardial fibrosis/necrosis by use of myocardial delayed enhancement. The delayed enhancement technique allows the detection of hyposignal areas (dark) amidst the hypersignal area (infarction), which relates to microvascular obstruction areas (no-reflow phenomenon), adding prognostic information for that population. In addition to those uses, CMR is extremely useful to differentiate ischemic from non-ischemic cardiomyopathies, being used to diagnose myocarditis and Takotsubo cardiomyopathy. Moreover, in the presence of elevation of myocardial necrosis markers and "normal" catheterization, CMR can confirm infarction, which could be related to spasm or thrombophilic syndromes^37^.

### Myocardial revascularization

The revascularization strategy (surgery or angioplasty) follows recommendations similar to those for patients with stable CAD. The major difference in the approach of patients with UA or NSTEMI is the greater benefit of early revascularization in those at higher risk for ischemic outcomes.

Patients with NSTE-ACS, especially those classified in the second tertile of the SYNTAX Score, should be assessed by the "Heart Team", and the decision about the type of revascularization, or even the isolated clinical treatment, should also consider circumstantial factors related to the experience of each center.

#### Myocardial revascularization surgery

The likelihood of complete revascularization is greater with CABG than with angioplasty. The benefit of CABG is greater in the subgroups of patients with diabetes or ventricular dysfunction.

The recently developed SYNTAX Score is a tool to support the revascularization strategy choice, because patients with a SYNTAX Score > 22 have better long-term results when submitted to CABG rather than to angioplasty^38^.

#### Percutaneous coronary intervention

In past decades, PCI has progressed, with greater experience of interventional cardiologists, better quality of the devices used (catheters, stents, balloons) and more effective and safe adjuvant drugs (antiplatelet and anticoagulant drugs). Such advances have allowed PCI indications to continuously and intensely increase in number, therefore promoting its use in more complex situations (left coronary artery lesions, multivessel disease and left ventricular dysfunction).

The major recommendations and respective levels of evidence in risk stratification and management in the first 12 hours from hospital arrival are shown in Tables 4 to 9.

**Table 4:** Risk stratification and management within 12 hours of hospital arrival

	**Clinical stratification**		
I (B)	All patients should be assessed and classified as high, intermediate or low probability of having NSTE-ACS		
I (B)	All patients with NSTE-ACS should be stratified and classified as at high, intermediate or low risk for developing major cardiac events. Classification by using more than one method is recommended, and the worst-case scenario should guide the decision on management		
I (B)	All patients with NSTE-ACS should be stratified and classified as at high, intermediate or low risk for developing bleeding		
	**Electrocardiography**		
I (C)	All patients with NSTE-ACS or suspected of having NSTE-ACS should undergo ECG. Ideally, ECG should be performed within 10 minutes of hospital arrival (level of evidence: B). ECG should be repeated in non-diagnostic cases at least once within 6 hours		
	**Necrosis biomarkers**		
I (B)	All patients suspected of having NSTE-ACS should have biomarkers of myocardial necrosis measured. The biomarkers should be measured on admission and repeated at least once 6-9 hours after (preferentially 9-12 hours after symptom onset) if the first measurement is normal or mildly elevated	IIb (B)	The CK-MB activity isolated or associated with total CK can be used if CK-MB mass or troponin are not available
I (A)	CK-MB mass and troponins are biomarkers of choice	IIb (B)	Myoglobin and high-sensitive troponin can be considered in association with a later marker (CK-MB or troponin) for patients who arrive early at the emergency unit (less than 6 hours from symptom onset)
		III (B)	Use of LDH, aspartate aminotransferase (GOT) or BNP/pro-BNP to detect myocardial necrosis in patients suspected of having NSTE-ACS
	**Exercise test**		
I (B)	Low-risk patients (clinic and ECG) with normal biomarkers should be referred for ET after 9 hours, ideally up to 12 hours, on an outpatient basis		
I (B)	When ET cannot be performed or when ECG cannot be interpreted, the patient can be stratified by using provocative test for ischemia with imaging		
I (B)	Treadmill or cycle ergometer protocols should be adapted to the clinical and biomechanical conditions of each patient		
	**Echocardiography**		
I (C)	Transthoracic echocardiography should be performed for the differential diagnosis with other diseases, when clinical suspicion of aorta diseases, pericardial diseases, pulmonary embolism and heart valve diseases exists	IIa (B)	In the presence of thoracic pain, patients can be assessed by using rest echocardiography to determine if the pain origin is ischemic or not
I (C)	In NSTE-ACS complications, such as ventricular septal defect and mitral regurgitation	IIa (B)	Patients with uncomplicated infarction of the anterior wall to determine the exact size of the ischemic injury
I (B)	Stress echocardiography is an alternative for patients unable to undergo ET		
	**Myocardial perfusion imaging**		
I (C)	Stress/rest myocardial perfusion imaging is an alternative for patients unable to undergo ET	IIa (A)	In the presence of thoracic pain, patients can be assessed by using rest myocardial perfusion imaging to determine if the pain origin is ischemic or not
	**Coronary computed tomography angiography**		
I (A)	To assess patients with acute chest pain at low to intermediate risk, with non-diagnostic ECG and negative markers of myocardial necrosis		

NSTE-ACS: Non-ST-elevation acute coronary syndromes; ECG: Electrocardiogram; ET: Exercise test.

**Table 9:** Secondary prevention

	**General guidance**
I (C)	Detailed instructions should be provided to patients with NSTE-ACS, including education on medications, diet and physical exertion, return to work and referral to a cardiac rehabilitation unit/secondary prevention program. Low-risk clinically treated and revascularized patients should have their first follow-up consultation in 2 to 6 weeks, and those at higher risk, within 14 days
	**Smoking cessation**
I (B) I (B)	Smoking cessation and no exposure to a smoking environment, at both work and home, are recommended. Long-term follow-up, referral to specific programs or drug therapy, such as nicotine replacement, are useful when associated with classical non-pharmacological strategies
	**Lipid approach**
I (C)	The lipid therapeutic approach should include assessing the fasting lipid profile of all patients within the first 24 hours from hospital admission
I (A)	For patients with NSTE-ACS and LDL-C ≥ 100 mg/dL, statins should be used unless contraindicated, aiming at reaching the LDL-C < 70 mg/dL goal

NSTE-ACS: Non-ST-elevation acute coronary syndromes.

## Figures and Tables

**Table 5:** Initial management of intermediate- and high-risk patients

	**Admission to coronary care unit**		
I (C)	All Intermediate- and high-risk patients with NSTE-ACS should be admitted to the coronary care unit until definitive management can be decided		
	**Oxygen therapy**		
I (C)	Oxygen therapy to intermediate- and high-risk patients (2 to 4 L/min) for 4 hours, or longer in the presence of desaturation < 90%		
	**Analgesia and sedation**		
I (C)	Administer morphine sulfate to intermediate- and high-risk patients	IIa (C)	Administer benzodiazepines to Intermediate-risk patients
I (C)	Administer benzodiazepines to high-risk patients Nitrates		
I (C)	Administer nitrate to intermediate- and high-risk patients		
	**Beta-adrenergic blockers**		
I (B)	Administer oral betablockers to intermediate- and high-risk patients	IIb (B)	Administer intravenous betablockers to intermediate- and high-risk patients
	**Calcium channel blockers**		
I (B)	Intermediate- and high-risk patients. Use non-dihydropyridine derivatives when betablockers are contraindicated	IIa (B)	Long-acting dihydropyridines in the presence of refractory ischemia for patients on proper use of nitrates and betablockers without ventricular dysfunction
		IIb (B)	Long-acting non-dihydropyridine derivatives as substitutes for betablockers, and short-acting dihydropyridine derivatives for high-risk patients already on proper use of betablockers
		III (B)	Short-acting dihydropyridine derivatives for patients not on proper use of betablockers
	**Inhibitors of the renin-angiotensin-aldosterone system**		
I (A)	ACEI for intermediate- and high-risk patients with left ventricular dysfunction, hypertension or diabetes mellitus	IIb (B)	ACEI to all intermediate- and high-risk patients
I (C)	Angiotensin receptor blockers for intermediate- and high-risk patients with contraindication to ACEI		

NSTE-ACS: Non-ST-elevation acute coronary syndromes; ACEI: Angiotensin-converting-enzyme inhibitors.

**Table 6:** Antiplatelet aggregation and anticoagulation in intermediate- and high-risk patients

	**Oral antiplatelet aggregation drugs**		
I (A)	ASA (162-300 mg loading dose, maintenance dose of 81-100 mg/day) to all patients, except when contraindicated, regardless of the treatment strategy, for undetermined time		
I (B)	Thienopyridine derivatives when ASA is contraindicated		
I (B)	Dual antiplatelet therapy for 12 months after the acute event, except when contraindicated		
I (A)	Clopidogrel (300 mg loading dose, maintenance dose of 75 mg/day) combined with ASA to intermediate- and high-risk patients with NSTE-ACS for 12 months	I Ia (B)	Clopidogrel (600 mg loading dose, followed by 150 mg/day for 7 days and then 75 mg/day) combined with ASA to patients undergoing PCI at high risk for ischemic events and low risk for bleeding
I (B)	Ticagrelor (180 mg loading dose, followed by 90 mg twice a day) to intermediate- or high-risk patients with NSTE-ACS, regardless of the following treatment strategy (clinical, surgical or percutaneous), for 12 months	I Ia (B)	Re-initiate ticagrelor, prasugrel or clopidogrel after CABG, as soon as safely possible
I (B)	Prasugrel (60 mg loading dose, followed by 10 mg/day) to intermediate- or high-risk patients with NSTE-ACS, with known coronary artery anatomy, treated with PCI, and with no risk factors for bleeding (age ≥ 75 years; < 60 kg; previous stroke or transient ischemic attack)		
		I Ib (B)	Use of platelet aggregability tests or genetic tests (genotyping) in selected cases
		III (C)	Combination of ASA with other non-steroidal anti-inflammatory drugs
	**Glycoprotein Ilb/IIIa receptor inhibitors - early interventional strategy**		
I (A)	Abciximab or tirofiban for high-risk patients when thienopyridine derivatives are chosen not to be administered		
I (B)	Addition of a GP IIb/IIIa inhibitor for patients at low risk for bleeding, on dual antiplatelet aggregation, undergoing high-risk PCI (presence of thrombi, thrombotic complications of the PCI)	III (A)	Routine use of GP I Ib/IIIa inhibitors to patients on dual antiplatelet aggregation before catheterization
	**Glycoprotein IIb/IIIa receptor inhibitors - conservative strategy**		
		I Ia (B)	Tirofiban to high-risk patients when thienopyridine derivatives are chosen not to be administered
		IIa (C)	Addition of GP I Ib/IIIa inhibitors for patients with recurrent ischemic symptoms during the use of oral dual antiplatelet aggregation and anticoagulation
		III (B)	Routine use of abciximab to high-risk patients
		III (A)	Routine use of GP I Ib/IIIa inhibitors to patients on dual antiplatelet aggregation before catheterization
	**Antithrombin agents**		
I (A)	UNH to all patients		
I (A)	Low-molecular-weight heparin to all patients	I Ia (A)	Use of enoxaparin rather than UNH, unless CABG is planned to occur within the following 24 hours
I (B)	Fondaparinux (2.5 mg, SC) once a day for 8 days or until hospital discharge	IIa (C)	Consider interrupting anticoagulation after PCI, unless otherwise indicated
I (B)	To patients on fondaparinux, administer UNH as follows: 85 lU/kg, IV, during PCI; or 60 IU/kg to those on GP I Ib/IIIa inhibitors	III (B)	Change of heparins (UNH and enoxaparin)

ASA: Acetylsalicylic acid; NSTE-ACS: Non-ST-elevation acute coronary syndromes; PCI: Percutaneous coronary intervention; UNH: Unfractionated heparin; CABG: Coronary artery bypass grafting

**Table 7:** Risk stratification with complementary tests for intermediate- and high-risk patients

	**Hemodynamic and cineangiocardiographic assessment**		
I (A)	Early hemodynamic and cineangiocardiographic assessment for intermediate- and high-risk patients	III (C)	Routine cineangiocardiography should not be indicated - even for intermediate/high risk patients - in the following situations: patients with important comorbidity and reduced life expectancy, or with no perspective on myocardial revascularization
	**Exercise test**		
I (B)	ET for intermediate-risk patients	lib (C)	ET performed in high-risk patients after 48 hours
		III (C)	ET performed in high-risk patients within 48 hours
	**Stress echocardiography**		
I (B)	Stress echocardiography for patients, about whom doubts remain after ET	IIa (B)	Stress echocardiography as an alternative to ET
		III (C)	Stress echocardiography for high-risk patients
	**Echocardiography with myocardial perfusion assessment**		
		IIa (B)	Contrast transthoracic echocardiography to improve Doppler signal in patients with suboptimal imaging, or to delineate endocardial margins during stress echocardiography in patients with suboptimal imaging at rest
		I Ib (B)	Stress echocardiography with microbubbles for intermediate-risk patients, about whom doubts remain after ET
		III (B)	Stress echocardiography with microbubbles for high-risk patients
	**Myocardial perfusion imaging**		
I (B)	Intermediate-risk patients, about whom doubts remain after ET, or unable to undergo ET	I Ib (B)	For intermediate-risk patients as the first stratification option
I (B)	To identify the presence/extension of ischemia in patients unable to undergo catheterization, or when the results of that test are insufficient to establish the management	III (C)	For high-risk patients before the first 48 hours of stabilization
I (A)	After catheterization to identify the event-related artery (region to be revascularized), and/or to perform complementary risk stratification		
I (A)	For patients with dyskinetic ventricular regions, requiring the confirmation or exclusion of viable myocardium to guide therapeutic approach		
	**Radionuclide ventriculography**		
I (A)	To assess left and right ventricular functions of intermediate- and high-risk patients	IIa (C)	To identify right ventricular impairment of intermediate- and high-risk patients
	**Cardiovascular magnetic resonance**		
I (A)	To assess ventricular function, presence/extension and viability of necrosis area	IIa (B)	In the differential diagnosis of patients with clinical findings compatible with acute coronary disease, but with unspecific ECG changes and negative biomarkers of necrosis
I (A)	To assess occasional mechanical changes	I Ib (B)	As an adjuvant in NSTE-ACS diagnosis, mainly in patients with intermediate or high likelihood

ET: Exercise testing; ECG: Electrocardiogram; NSTE-ACS: Non-ST-elevation acute coronary syndromes.

**Table 8:** Myocardial revascularization

	**Complex coronary artery disease**		
I (C)	Multidisciplinary heart team (clinician, surgeon and specially trained cardiovascular physician)		
I (B)	Knowledge about the patient's surgical risk (institution's score and/or STS Score and/or Euroscore)		
I (B)	Knowledge about coronary artery anatomy (SYNTAX Score)		
	**Lesion in the left main coronary artery**		
I (B)	CABG	IIa (B)	Angioplasty: if the patient has a high-risk for surgery or unstable angina or NSTEMI and is not candidate for surgery
	**Three-vessel disease with or without lesion in the anterior AD**		
I (B)	CABG	IIa (B)	Surgery provides more benefit than angioplasty, if SYNTAX Score > 22
		IIb (B)	Angioplasty
	Two-vessel disease with proximal lesion in the AD		
I (B)	CABG	IIa (B)	Angioplasty
	**Two-vessel disease without proximal lesion in the AD**		
		IIa (B)	CABG using internal thoracic artery
		IIa (B)	Angioplasty
	**Single-vessel disease without proximal lesion in the AD**		
I ( )	Angioplasty with large myocardial area at risk	III ( )	CABG
	**Revascularization strategy to relieve angina**		
I ( )	Angioplasty or CABG if one or more vessels are impaired and angina persists despite optimized clinical treatment	IIa ( )	Surgery preferred over angioplasty for patients with complex multivessel disease with or without proximal AD (SYNTAX Score > 22)
		III ( )	Coronary arteries lacking anatomical conditions for revascularization or no ischemia

CABG: Coronary artery bypass grafting; AD: Descending artery; NSTEMI: ST-segment elevation.
